# XAFS Data Interchange: A single spectrum XAFS data file format

**DOI:** 10.1088/1742-6596/712/1/012148

**Published:** 2016-06-06

**Authors:** B. Ravel, M. Newville

**Affiliations:** 1 National Instituts of Standards and Technology Gaithersburg, MD 20899, USA; 2 Center for Advanced Radiation Studies, University of Chicago, Chicago, IL 60637, USA

## Abstract

We propose a standard data format for the interchange of XAFS data. The XAFS Data Interchange (XDI) standard is meant to encapsulate a single spectrum of XAFS along with relevant metadata. XDI is a text-based format with a simple syntax which clearly delineates metadata from the data table in a way that is easily interpreted both by a computer and by a human. The metadata header is inspired by the format of an electronic mail header, representing metadata names and values as an associative array. The data table is represented as columns of numbers. This format can be imported as is into most existing XAFS data analysis, spreadsheet, or data visualization programs. Along with a specification and a dictionary of metadata types, we provide an application-programming interface written in C and bindings for programming dynamic languages.

## 1. The problem of XAFS data representation

A poorly structured and poorly documented file format hinders the use of the data contained in the file by limits its portability between computers, between data analysis and data visualization programs, and over time. What's more, data contained in a poorly formatted file is ill-suited for deposition with a journal as supplemental material.

Virtually every XAFS beamline in the world provides some kind of single-scan file format to its visitors. All such files capture the data table associated with the measurement in some manner. Many capture some metadata relevant to the provenance and measurement conditions of the data. The representation of the metadata and the data table is wildly inconsistent from beamline to beamline. Indeed, there is not even agreement as to a standard representation of the abscissa of the data, with extant examples of the abscissa represented in units of eV, keV, monochromator angle, and photon wavelength.

We propose a solution to the problem of representing a single spectrum of XAFS data called “XAFS Data Interchange” or XDI. Our goals for XDI include:

1. Establish a common format for transferring data between XAS beamlines, XAS experimenters, data analysis packages, web applications, and anything else that needs to process XAS data.
2. Increase the relevance and longevity of experimental data by reducing the amount of data archaeology future interpretations of that data will require.
3. Enhance the user experience by promoting interoperability among data acquisition systems, data analysis packages, and other applications.
4. Provide a mechanism for extracting and preserving a single XAS-like data set from a related experiment (for example, a resonant diffraction or inelastic scattering measurement) or from a complex data structure (for example, a database or a hierarchical data file used to store a multi-spectral data set).
5. Provide a representation of an XAS spectrum suitable for deposition with a journal or into a database.

In short, we need to share data across continents, decades, and analysis toolkits.

XDI is designed to capture a *μ*(*E*) spectrum as an ASCII column data file. While it may, in certain situations, be a suitable format for raw data from a beamline, it cannot possibly capture the full complexity of a modern synchrotron measurement. For example, it is not suitable for multi-dimensional or multi-spectral measurements such as a microspectroscopy image. It is not intended to represent a fluorescence measurement made with a large number of energy-discriminating detector elements. To fully capture the data from an energy-discriminating detector requires at least three numbers per channel at each energy point. This would make an unmanageably wide data table when represented as a column text file. It is also not intended to fully encapsulate the path of a measurement through data processing and analysis. There exist hierarchical, relational, and compressed file formats which would be more suitable for any of those chores.

The intent, then, of XDI is to represent a single, processed *μ*(*E*) spectrum. This might be a single scan, or the average of repeated scans on a sample, or the consolidation of the channels of a multi-element detector after dead-time corrections have been applied. In any case, a processed *μ*(*E*) spectrum is the basic unit of currency for an XAFS analysis problem and an XDI file is the encoding of this basic unit of currency.

## 2. The XAFS Data interchange format

**Figure 1:**
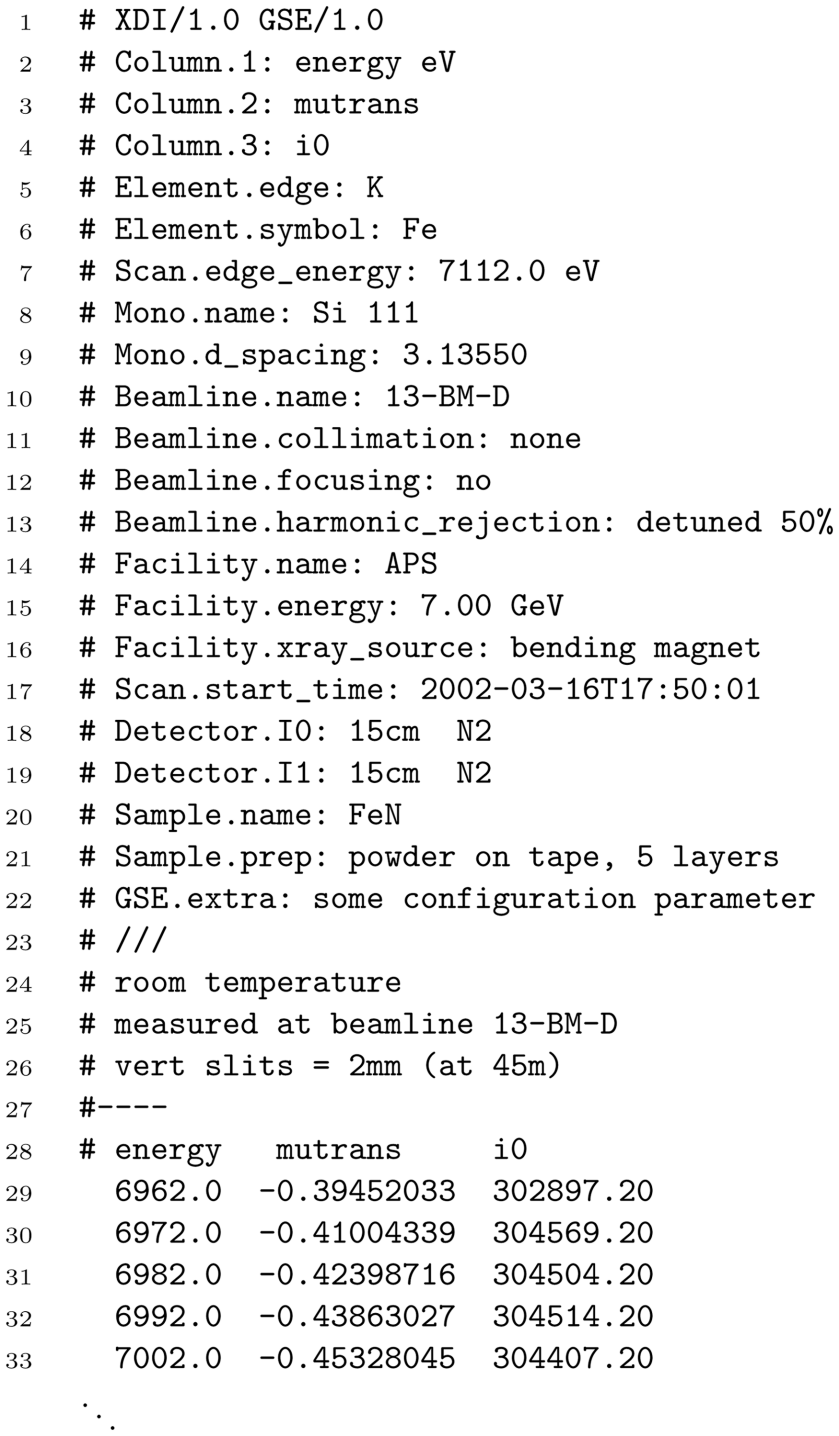
Listing of an XDI file containing transmission XAFS data measured on iron nitride, FeN. The entire header and the first five data points are shown. The data table has columns for the energy axis, *μ*(*E*), and the signal on the incident intensity detector and it continues to the end of the file.

Figure 1 shows an example of a transmission XAFS spectrum represented in the XDI format. There is a clear separation between the parts of the file which contain metadata and the data table. The file begins with the metadata portion. Every line of the metadata portion begins with a hash (#) character. After the metadata portion, the data table is represented as columns of integer or floating point numbers.

The XDI file format and the version number of the specification used to generate the file are given in line 1. Lines 2–21 show various examples of metadata. A line containing metadata starts with a hash character, followed by a two-part name, followed by a colon (:). The two parts of the metadata name represent a semantic grouping, or *family*, and a keyword. For example Mono.d_spacing at line 9 is in the family of metadata pertaining to the monochromator and contains the *d*-spacing of the crystal used to monochromate the incident light. The value, in this case 3.13550 Å, follows the colon.

This way of representing the metadata maps naturally onto the concept of an associative array, with the colon separating the key/value pair. The definition of semantic families, like Mono, allows a mapping of metadata onto nested associative arrays, useful for programming languages that support nested data structures.

Information about the data table – including number of columns, column labels, and units on the abscissa – are shown in lines 2–4. It is good practice to replicate the column labels in the last header line preceding the data table, as shown at line 28.

Three items of metadata are *required* in any compliant XDI file, Mono.d_spacing, Element. symbol, and Element. edge (seen at lines 5, 6, and 9). The monochromator *d*-spacing is required if a correction needs to be made to the energy axis of the data table. The atomic and edge symbols of the absorbing element are required to unambiguously identify the data. For example, both Cr K and Ba L_I_ have tabulated edge energies of 5989eV, while the Se K and Tl L_III_ edges are both at 12658 eV.

Five items of metadata are *recommended* to be included in any XDI file. Four of them – Beamline.name, Facility.name, Facility.xray_source, and Scan.start_time (lines 10, 14, 16, and 17) – capture the most essential details of the provenance of the data. Column.1 (line 2) unambiguously identifies the energy units of the abscissa.

XDI allows the capturing of metadata outside the dictionary through the use of *extension fields*. An example is shown at line 22. The letters “GSE” refer to the data acquisition system at the beamline where these data were measured. Any data acquisition, processing, or analysis program can add metadata to the XDI file header. First the application identifies itself in line 1 after the string which identifies the XDI version number. Using the same identifier string as the family part of the metadata label, the application appends metadata lines using the same syntax as for dictionary metadata lines. In this way, any application which touches the XDI file can encode its metadata into the file.

There is a space for user-supplied comments, as seen at lines 24-26. The metadata section of the header ends at line 23 with text that contains the hash character followed by three or more forward slash characters (/). This is followed by optional, freely-formatted text, then terminated at line 27 with text that contains the hash character followed by three or more dash characters (–). This comment text is most commonly used at the time of data acquisition by the beamline visitor. This text is free-form and there is no expectation that it should be interpreted by any later application.

Finally, the data table is simply columns of numbers extending to the end of the file. Every entry in the data table must be interpretable as a number. The leftmost column of the data table must contain the abscissa in the units defined in the Column.1 metadata line. Each row of the data table must have the same number of columns.

## 3. The XDI implementation

All work related to XDI is hosted as a GitHub repository at https://github.com/XraySpectroscopy/XAS-Data-Interchange. The documentation at the repository includes a full specification of the XDI format and a dictionary of metadata defining several meaningful concepts related to the provenance and measurement conditions of a spectrum. The specification includes complete details for what constitutes a valid metadata name string or a valid numeric entry in the data table.

The specification is implemented as a library written in the C programming language. This library reads and interprets the contents of the XDI file in the following steps:

1. Parse the XDI file, stopping only in the case of an unrecoverable error, then
2. Check that the required metadata, as identified in the specification, are present, then
3. Check that the recommended metadata, as identified in the specification, are present, then
4. Validate each individual metadata item against its dictionary definition, if available

The library returns a data structure containing the full content of the XDI file. The entire content of the specification and the dictionary are encoded in the library.

This library is easily bound to dynamic languages. Bindings are provided at this time for Python and Perl. New bindings for other languages can be created using the C library. A less fully-featured implementation is provided in the FORTRAN language.

The distribution also includes a cross-platform build system, documentation, an extensive test suite of compliant and non-compliant data files, and automation for testing the library.

XDI is available for use. Participation in this effort via the GitHub site is encouraged. We hope to see wide adoption of XDI at beamlines, in programs for data processing and analysis, in XAS theory programs, and elsewhere.

